# HCG-responsive aldosteronoma with transient secretion during pregnancy confirmed through HCG-stimulated adrenal venous sampling

**DOI:** 10.3389/fendo.2023.1153374

**Published:** 2023-02-28

**Authors:** Frederic Castinetti, Carole Guerin, Estelle Louiset, André Lacroix

**Affiliations:** ^1^ Department of Endocrinology, Aix Marseille University, Assistance Publique-Hopitaux de Marseille, INSERM, Marseille Medical Genetics, Marmara Institute, La Conception Hospital, Marseille, France; ^2^ Aix Marseille University, Assistance Publique Hopitaux de Marseille, Department of Endocrine Surgery, La Conception Hospital, Marseille, France; ^3^ Univ Rouen Normandie, INSERM, NORDIC UMR 1239, Rouen, France; ^4^ Division of Endocrinology, Department of Medicine, Research Center, Centre hospitalier de l’Université de Montréal (CHUM), Montréal, Québec, QC, Canada

**Keywords:** aldosteronoma, LHCG receptor, aberrant G protein coupled receptor, pregnancy, primary aldosteronism

## Abstract

Primary aldosteronism can be regulated by the ectopic expression of G-protein coupled receptors in aldosteronomas or bilateral hyperplasias. We report a rare case of a young woman in whom 2 pregnancies were complicated by pre-eclampsia and 1 miscarriage. The transient primary aldosteronism during pregnancies suggested the possibility of HCG stimulated aberrant adrenal expression of LHCG receptor in her adrenal tissues. This was supported by increased aldosterone and renin suppression during 5-day HCG stimulation test outside of pregnancy. Following a second 5-day HCG stimulation test, bilateral simultaneous adrenal vein sampling identified a lateralized source of aldosterone from an 8 mm right adrenal nodule. A right laparoscopic adrenalectomy resulted in clinical and biochemical cure and allowed a further uneventful pregnancy a few years later. This case illustrates the indication to investigate for potential primary aldosteronism in woman with transient hypertension during pregnancy.

## Introduction

Primary aldosteronism is increasingly recognized as the most frequent cause of secondary hypertension, responsible for up to 20% of patients with resistant hypertension (1). This still largely underdiagnosed disease is secondary to two main presentations, with aldosterone secreting lateralized adenoma or bilateral adrenal hyperplasia (in 50-70% of cases). The precise pathophysiological triggering mechanisms leading to increased aldosterone secretion remain incompletely understood, but mutations of several somatic ion-channel genes are found in aldosteronomas and micronodules (1). In physiology, but also in disease, ACTH can regulate aldosterone secretion depending on the levels of its G-protein coupled receptor (GPCR) called MC2R (2). Aberrant expression of other GPCR can also be involved in aldosterone excess: GPCR can be eutopic (5-HT4R for serotonin), or ectopic (such as the glucose-dependent insulinotropic peptide receptor, beta-adrenergic receptor, vasopressin receptor or glucagon receptor) (2). LH/HCG GPCR (LHCGR) and GnRHR can also be ectopically expressed in adrenal adenomas or adrenal hyperplasia leading to hyperaldosteronism (1). Roughly 15 years ago, Zwermann et al. reported that the majority of both normal (though at low amounts) and tumoral adrenal tissues (14 aldosterone producing adenomas) expressed LHCGR mRNA; however, only 3 were responsive to GnRH stimulation (3). The same team identified GnRHR expression in 4/15 (26.7%) adrenal adenomas or hyperplasia leading to primary aldosteronism, concordant with the previous work of Saner-Amigh et al., showing GnRHR expression in 11/28 (39.3%) adrenal producing adenomas (4). In 2011, Albiger et al. reported for the first time a diagnosis of primary aldosteronism during pregnancy due to the ectopic expression of LHCGR in an adrenal adenoma (5). They also described GnRH response *in vivo* in 83% of 12 patients with primary aldosteronism (3 positive and 7 partial responses). This original case was followed by the description of 3 cases with primary hyperaldosteronism during pregnancy (n=2) and menopause (n=1), showing LHCGR and GnRHR overexpression in adrenal adenomas by microarray analysis and qPCR. The clinical demonstration of regulation of aldosterone *in vivo* was however limited in these 3 cases (6). Aldosterone was aberrantly regulated by various stimuli in ~85% of 43 patients with lateralized or bilateral source of primary aldosteronism, including response to GnRH or recombinant LH tests in approximately 50% of them (7).

We report the exhaustive investigation of a woman with HCG-responsive primary aldosteronism diagnosed during pregnancy. We performed an HCG-stimulated adrenal venous sampling to confirm the lateralized nature of the disease to allow the appropriate surgical management. Surgery was curative as shown by a post-operative HCG challenge stimulation test, and further uneventful pregnancy. The patient gave her written informed consent for publication of the case.

## Case presentation

### Diagnostic assessment

The patient had a first pregnancy in 2007 at the age of 23 which was complicated by hypertension with pre-eclampsia and hypokalemia (2.7 mmol/l), requiring a caesarean section. The patient reported no previous history of hypertension nor hypokalemia. She had a second pregnancy in 2011 which terminated with spontaneous miscarriage at 7 weeks of gestation in the context of a severe hypokalemia (1.9 mmol/l) with increased blood pressure (155/100 mm Hg). Because of this history of hypertension and hypokalemia during pregnancy, an abdominal CT scan revealed a solid 8 mm right adrenal nodule with a spontaneous density of less than 10 HU and a left adrenal thickening. Hormone measurements performed 3 months after miscarriage did not reveal any abnormalities (normal methoxytyramines; renin 4,9 mIU/L - normal range 4.5-40; aldosterone 233 pmol/l – normal range 86-1080; overnight dexamethasone suppression test cortisol, 32 nmol/l). Blood pressure and kalemia were normal between each pregnancy. The patient had a third pregnancy in 2013 with hypertension and hypokalemia beginning from the 5th week. Treatment with low-dose spironolactone and alpha methyldopa was initiated but did not prevent proteinuria and edema of the lower limbs during the third trimester, requiring premature delivery after 32 weeks of gestation. The patient was referred to us 6 months later with suspicion of primary aldosteronism due to ectopic expression of G-protein coupled LHCGR in the adrenocortical tissue. The patient clearly desired future pregnancies. On clinical examination, 6 months after delivery, the BMI was 25 kg/m2, repeated blood pressure measurements were normal (mean, 114/55 mm Hg). Potassium level was normal (3.8 mmol/l). An HCG stimulation test was carried out (5000 IU HCG daily with intra-muscular injection for 5 days): the test showed an abnormal response of renin and aldosterone (**Table 1** before surgery). More specifically, on day 5, renin was undetectable, aldosterone was increased and associated with hypokalemia. No GnRH test was performed.

**Table 1:** HCG stimulation tests before and after adrenal surgery.

	HCG	Potassium	Urinary potassium	Renin	Aldosterone
Before surgery					
**D1 T0+0.5h**	14	3.7	NA	3.3	69
**D1 T0+6h**	52	3.7	30	2.9	86
**D2 T0+6h**	105	3.5	30	2.8	698
**D3 T0+6h**	146	3.3	50	3.8	903
**D4 T0+6h**	241	3.2	61	3.0	1382
**D5 T0+6h**	349	3.1	124	<1.8	1343
After surgery					
**D1 T0+0.5h**	14	4.7	NA	21	66.5
**D1 T0+6h**	34	4.4	NA	42	133
**D2 T0+6h**	86	4.3	21.2	43.4	219
**D3 T0+6h**	151	4.5	25.5	39.5	142
**D4 T0+6h**	249	4.8	29.4	56	241
**D5 T0+6h**	266	4.4	44.2	58.5	235

Plasma HCG (IU/L), potassium (mmol/l), renin (mIU/L) and aldosterone (pmol/l, normal range 86-1080) concentrations and urine potassium (mmol/l) concentrations measured after daily injections of HCG (5000 IU) in the patient before and after surgery. D1 stands for the first day of injection, D2 for the second … and +xh stands for the time the sampling was taken after HCG injection. NA, not available.

As the patient was presenting with a unilateral adrenal nodule and a slightly hyperplastic contralateral adrenal gland, we performed a bilateral simultaneous intravenous adrenal venous sampling (AVS) on day 5 of a second HCG stimulation test (without any exogenous ACTH administration). Peripheral blood sample after 5 days of HCG stimulation showed undetectable renin (< 0.2 mIU/L) and increased aldosterone (1421 pmol/l; normal range, 86-1080) confirming the previous response. The bilaterally selective AVS (Selectivity ratio > 5 bilaterally) showed a right lateralized source of aldosterone (right/left lateralization ratio, 18.1) and adequate direct contralateral aldosterone ratio (left vein/periphery) of 1.7 under HCG stimulation (normal value < 2) (**Table 2**).

**Table 2:** Bilateral simultaneous adrenal vein sampling performed on day 5 of HCG stimulation.

	Aldosterone pmol/l	Cortisol nmol/l	Aldosterone/ Cortisol ratio	Lateralization ratio right to left
**Vena cava**	1246	757		
**Right adrenal vein**	29528	5813	5.07	
**Left adrenal vein**	2105	7500	0.28	18.1

### Laparoscopic adrenalectomy and histopathological studies of resected aldosteronoma

**Figure 1:**
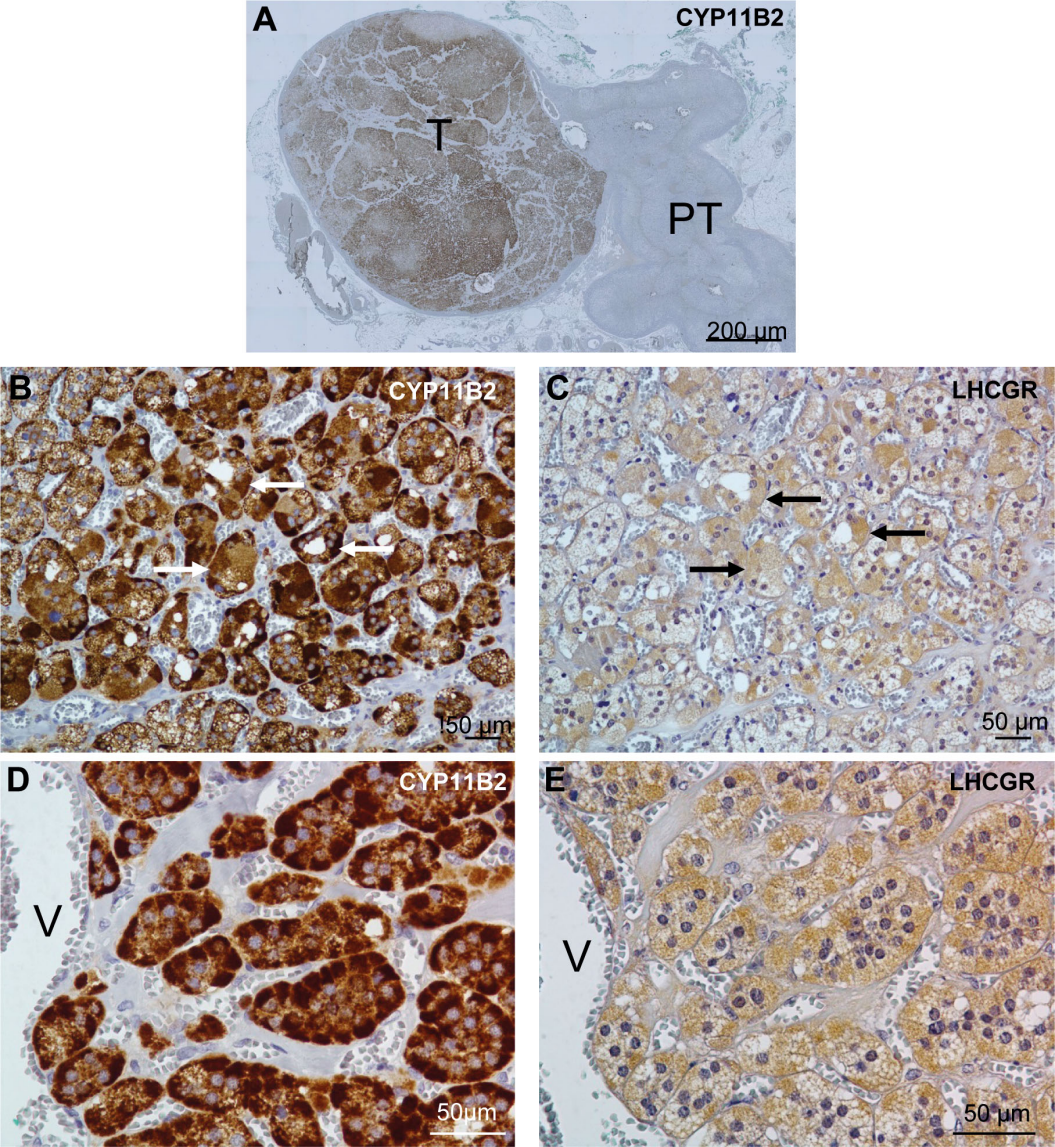
Distribution of aldosterone synthase (CYP11B2) and LHCGR in the patient’s adrenal. **(A)**, Distribution of CYP11B2 immunoreactivity in the tumor region (T) of the adrenal tissue. **(B-E)**, Distribution of CYP11B2 **(B, D)** and LHCGR **(C, E)** immunoreactivities in consecutive sections of the APA tissue at low **(B, C)** and high **(D, E)** magnifications. Similar distribution of CYP11B2 and LHCGR immunoreactivities were observed in some areas (arrows) in **(B, C)**. PT indicates peritumoral tissue; and V, vein.

The patient underwent a right laparoscopic adrenalectomy. Pathology confirmed a benign adrenocortical nodule (Ki67 < 1%) without adjacent adrenal hyperplasia. Immunohistology was performed on formalin-fixed and deparaffinized tissue sections. Before CYP11B2 immunostaining, sections were heated at 95 °C for 20 minutes in Tris-EDTA buffer pH 9 for antigen retrieval. The slides were incubated with peroxidase blocking reagent (Dako-Agilent, Les Ulis, France), and saturated with 5% normal goat serum for 20 min. Sections were then incubated for 60 min at room temperature or overnight at 4 C with CYP11B2 (clone 41-17B at 1:500; Millipore) or LH-R (PA-1552 at 1:100; Boster) antibodies, respectively. Immunoreactivities were revealed with anti-immunoglobulin coupled to peroxidase and diaminobenzidine (Dako-Agilent). Tissue sections were counterstained with hematoxylin and identified the right adenoma. Strong CYP11B2 staining in the adenoma confirmed aldosterone source from this adenoma without any positive adjacent nodular hyperplasia (**Figure 1**). Complementary immunochemistry revealed positive LHCGR staining in small steroidogenic cells within the aldosteronoma (**Figure 1**).

### Outcome and follow-up

Three months after the right adrenalectomy, the patient underwent a third test with HCG stimulation, which did not reveal any increase in aldosterone, nor hypokalemia or renin decrease (**Table 1** after surgery). The patient was again pregnant in 2020, without any comorbidity such as hypertension or hypokalemia during pregnancy.

## Discussion

This case demonstrates transient and repeated episodes of primary aldosteronism during pregnancies secondary to the ectopic expression of LHCGR in the patient’s aldosteronoma. The originality of the report is enhanced by the use of HCG stimulation test to confirm stimulation of aldosterone response outside of pregnancy and during AVS to confirm the source of aldosterone excess from the right adrenal adenoma. Post-surgical normal response to HCG, and further pregnancy confirmed that surgical resection of the aldosteronoma cured the patient. More than 10 years ago, Albiger et al. reported the first case of a 32-year-old female with hypertension after 28 weeks of pregnancy, with primary aldosteronism due to ectopic expression of LHCGR and GnRHR in a left adrenal nodule: shortly after delivery, as she still had primary hyperaldosteronism, AVS was performed indicating aldosterone hypersecretion on the left side, indicating a left adrenalectomy (5). In contrast to our case, AVS was performed without stimulation with HCG.

As only rare cases of LH/HCG stimulated hyperaldosteronism were reported, the pathophysiological mechanisms are not fully understood. Teo et al. reported in 2015 the occurrence of heterozygous pathogenic variants in exon 3 of the β-catenin gene (*CTNNB1)* in 3 adenomas of women with hyperaldosteronism during pregnancy or menopause. The hypothesis of the authors was that these mutations could modify the fate of an adrenal cell, switching it to a cell also expressing gonadal genes, thus leading to the expression of LHCGR and GnRHR. In their study, primary zona glomerulosa-like adenoma cells transiently transfected with the *CTNNB1* mutations found in the patients induced the expression of LHCGR. However, no further testing with GnRH showing aldosterone response was performed (6). The involvement of somatic *CTNNB1* mutations in the induction of LHCGR and GnRHR ectopic expression and pregnancy-LH/HCG stimulated primary aldosteronism has been questioned. While several studies reported a relatively high incidence of aldosterone response to GnRH or LH *in vivo* (7), less than 10% mutations of *CTNNB1* have been reported in unselected aldosterone-producing adenomas (8). Gagnon et al. did not find any *CTNNB1* mutation in 11/23 patients with positive or partial aldosterone response to GnRH or LH test *in vivo*; in contrast, 2 patients with *KCNJ5* mutation also showed a positive response to GnRH *in vivo* (9). More recently, overexpression of LHCGR required combined mutations of *GNA11* and *GNAQ* in *CTNNB1*-mutant aldosterone-producing adenomas presenting in puberty, pregnancy or menopause (10). While the Wnt pathway seems crucial for adrenal development of the zona glomerulosa, it remains thus uncertain if mutations of *CTNNB1* specifically plays a role in HCG-responsive primary aldosteronism. In GIP-dependent primary bilateral macronodular adrenal hyperplasia with Cushing’s syndrome, *KDMIA* mutations were found to be responsible for ectopic GIPR expression but could also result in increased expression of LHCGR (11). Unfortunately, such a somatic genetic analysis could not be performed in our patient’s aldosteronoma.

The relatively rapid response to exogenous HCG stimulation outside of pregnancy confirms that the aberrant LHCGR remains capable of rapid activation, as demonstrated also by the rapid appearance of hypertension and hypokalemia at each pregnancy; this is similar to the transient Cushing’s syndrome during pregnancy when LHCGR is expressed in primary bilateral macronodular adrenal hyperplasia (PBMAH) patients (12). More recently, it was shown that life-threatening adrenergic myocarditis during pregnancy resulted from ectopic expression of LHCGR in a pheochromocytoma in which placental HCG stimulated epinephrine secretion (13).

The impact of primary hyperaldosteronism on pregnancy requires an optimal management before pregnancy. A recent case control study based on 20 women with primary aldosteronism compared their outcome during pregnancy with 20 women with high or low-risk hypertension. Before surgery, the number of anti-hypertensive treatments was similar between primary aldosteronism and high-risk groups, while the number increased more in patients with primary aldosteronism during pregnancy. While the rate of pre-eclampsia was similar between the 2 groups, the hospital stay was longer in women with primary aldosteronism. This emphasized the need for a proper screening of women with hypertension before pregnancy (14). Concordant with this, during her first 3 pregnancies, our patient had 2 pre-eclampsia episodes and one spontaneous miscarriage before the diagnosis was made. The rare cases reported in the literature do not allow to conclude that LH/HCG stimulated primary aldosteronism could lead to a more severe hypertensive phenotype than other causes of primary aldosteronism during pregnancy. Irrespective of the GPCR status, Amar et al. reviewed 31 cases of primary aldosteronism diagnosed during pregnancy: blood pressure was uncontrolled in a third of them despite medical treatment, and 16% (n=6) required adrenalectomy during pregnancy because of uncontrolled hypertension; 5 patients had preeclampsia and 19 had induced delivery (mean term was 33.7 weeks of gestation). Only one early miscarriage (before 8 weeks) was reported in the literature (15).

To conclude, this case illustrates a rare presentation of transient and repetitive LH/HCG stimulated primary aldosteronism revealed during several pregnancies. While we did not perform a GnRH stimulation test, we performed an original 5-day HCG stimulation test outside of pregnancy, which provoked a sustained aldosterone response, and allowed identifying the lateralized right source of aldosterone excess (and not from possible left adrenal hyperplasia), during adrenal vein sampling performed under HCG stimulation. This allowed us to propose unilateral adrenalectomy in this young patient who desired to have a further pregnancy. The story of our patient with 2 complicated pregnancies and one early miscarriage also emphasizes the need for screening young patients with hypertension for primary aldosteronism before pregnancy, to propose an optimal therapeutic approach. Finally, even though genetic somatic testing could not be performed in our patient, further studies should be aimed at trying to better understand the pathophysiological mechanisms leading to the expression and activation of ectopic LHCGR in primary aldosteronism.

## Learning points

- Primary aldosteronism can be regulated by aberrant overfunction of G protein coupled receptors

- Ectopic adrenocortical expression of LHCGR and GnRHR can lead to transient severe hyperaldosteronism during pregnancy or become progressive after menopause

- The pathophysiological mechanisms leading to the aberrant expression of LHCGR and GnRHR remain incompletely understood.

- GnRH and/or HCG stimulation test should be systematically performed in young women with hypertension/hypokalemia confined to pregnancy ((suspicion of transient primary aldosteronism) to look for *in vivo* response of aldosterone.

## Author contributions

All authors made individual contributions to authorship. FC was involved in the diagnosis and management of this patient and initial manuscript writing and submission. CG: responsible for the patient’s surgery EL: histopathology section and preparation of histology images. AL was involved in manuscript writing and submission. All authors contributed to the article and approved the submitted version.
